# Existential concerns, community integration and psychological depression among female stroke survivors in Nigeria

**DOI:** 10.3389/fstro.2025.1635705

**Published:** 2025-10-14

**Authors:** Olubukola A. Olaleye, Ayomide T. Olajide

**Affiliations:** Department of Physiotherapy, College of Medicine, University of Ibadan, Ibadan, Nigeria

**Keywords:** female, stroke survivors, community reintegration, existential concerns, emotional and mental wellbeing, depression

## Abstract

**Introduction:**

Female stroke survivors experience considerable vulnerabilities and existential concerns, shaped by sociocultural factors and gender roles, which heighten stroke morbidity and limit community reintegration. Yet, the existential concerns of female stroke survivors in Nigeria, and their relationships with psychological depression and community reintegration have not been explored.

**Methods:**

A mixed-methods study was conducted among female stroke survivors recruited from selected hospitals in South-west, Nigeria. Community integration questionnaire, Existential concerns questionnaire, and the depression subscale of the Hospital anxiety and depression scale were used to assess community reintegration, existential concerns, and psychological depression, respectively. Six purposively selected female stroke survivors participated in a focus group discussion (FGD). Quantitative data were analyzed using Chi-square test at *p* < 0.05, while qualitative data were thematically analyzed.

**Results:**

Seventy-five female stroke survivors aged 64.07 ± 14.03 years participated in the survey. The mean community reintegration, existential concerns and psychological depression scores were 12.24 ± 2.95, 9.77 ± 5.52, and 13.84 ± 4.71, respectively. The majority (*n* = 61; 81.3%) of the participants had a low level of community integration. Forty-seven (62.7%) reported a moderate level of existential concerns, while 32(42.7%) had psychological depression. There was a significant association between community reintegration and psychological depression (*p* = 0.02), and between existential concerns and psychological depression (*p* < 0.01). However, there was no association between community reintegration and existential concerns (*p* = 0.08). The five emergent themes from the FGD were: perception of stroke as a devastating condition; role disruption and loss of autonomy in the home, isolation and stigmatization in society, inadequate spousal support and sexual intimacy, work-related and financial concerns.

**Conclusion:**

Existential concerns among participants were mostly related to social and family roles and were associated with poor emotional and mental wellbeing. Addressing these concerns through integrated care, delivered by a coordinated multidisciplinary team, could enhance emotional and mental wellbeing, and promote community reintegration among female stroke survivors.

## 1 Background

Stroke is the third leading cause of death and a major cause of disability among women (Petrea et al., 2009). Female stroke survivors often experience worse functional outcomes than males, attributable to older age at onset, poorer pre-stroke functional and health status, higher comorbidity rate, limited social support, and greater dependency in activities of daily living (Liljehult et al., 2021; Xu et al., 2022; Zhou et al., 2024; Rizzo et al., 2024). These vulnerabilities extend beyond physical functioning to psychosocial and existential concerns, with female stroke survivors facing lower levels of community reintegration, heightened risks of depression and unique gender-related challenges (Hamzat et al., 2014; Witt et al., 2024). The objectives of this study were to explore the existential concerns of female stroke survivors in Nigeria, and to investigate the associations among community reintegration, existential concerns and psychological depression. We hypothesized that female stroke survivors with greater existential concerns would exhibit higher levels of psychological depression and lower levels of community reintegration.

Community reintegration encompasses important aspects of post-stroke life, including leisure and social participation, economic and residential integration, employment stability, familial roles, coping mechanisms and independent living (Akosile et al., 2016). It is an important component of stroke recovery However, inadequate support systems, isolation and restrictive gender expectations often impede women's reintegration into the community, deepening the impact of stroke beyond physical disability to existential concerns (Walsh et al., 2015; Thompson and Ryan, 2019).

Existential concerns, such as death, isolation, loss of identity and autonomy, and the search for meaning, can interfere with mental health, exacerbate loneliness and intensify uncertainty (Kretschmer and Storm, 2018; Vail et al., 2020). Many female stroke survivors struggle with role reversal trauma as they transition from caregivers to care recipients, a process worsened by cultural expectations and traditional gender roles (Nettles, 2024; Pathan et al., 2024; Wan et al., 2024). These concerns heighten psychological distress, as women worry about loss of control and diminished autonomy (Agbola et al., 2020), and have been associated with post-stroke depression (PSD) (Kingau et al., 2024).

Depression is a common and debilitation complication of stroke. Dymm et al. (2024) reported that 31.7% of women with small vessel stroke experienced PSD compared to 6.3% of controls. Evidence suggests high rates of depressive symptoms among female stroke survivors in Nigeria, particularly those with severe disabilities and post-stroke complications (Bakare et al., 2024). Female gender itself has been identified as an important risk factor for PSD (Harini and Suraweera, 2023), alongside low level of education, dissatisfaction with life, poor social support and pain (Xiao et al., 2024). PSD interferes with recovery by worsening physical functioning, quality of life, and survival (Shewangizaw et al., 2023). It also impairs concentration, motivation and energy levels, further hindering reintegration (Argyriadis et al., 2020; Terrill, 2023).

Despite extensive research on the physical impacts of stroke, the psychological and existential dimensions remain underexplored, particularly among women in low-and middle-income countries (Dahlby and Boyd, 2024). Moreover, female stroke survivors are underrepresented in stroke research, limiting the development of gender-sensitive rehabilitation strategies that reflect their unique experiences (Dahlby and Boyd, 2024). In Africa, where socio-cultural expectations amplify vulnerabilities, women face peculiar existential challenges that require culturally appropriate, gender-responsive support systems (Santiago, 2024). Addressing these issues is essential to improving clinical outcomes and the overall wellbeing of Nigerian female stroke survivors.

## 2 Methodology

### 2.1 Study design

A concurrent mixed-methods research design; comprising a cross-sectional survey and a focus group discussion was adopted for this study. This approach provided broad and complementary insights into the existential concerns of the participants. The survey was used to obtain quantitative data while the focus group discussion elicited qualitative data on the personal experiences and existential concerns of participants.

### 2.2 Participants

A convenience sample of female stroke survivors recruited from the physiotherapy clinics of the four hospitals in South-west Nigeria (University College Hospital, Oyo state; Ring Road State Hospital, Oyo state; Obafemi Awolowo University Teaching Hospital, Osun state; and University of Osun Teaching Hospital, Osun state), participated in the survey. Six participants from this sample were purposively recruited for the focus group discussion. Eligible participants were: adults aged 18 years and above, with first incident stroke and a minimum post-stroke duration of 3 months, and who could comprehend and communicate freely in English and/or Yoruba language. Comprehension was determined by the participants' ability to follow a three-step command (indicating minimal or no cognitive impairment) (Olaleye et al., 2014). Female stroke survivors with co-existing neurological conditions such as Parkinson's disease and dementia; and those who had undergone mastectomy or any similar intervention that could lead to or had been associated with psychological distress were excluded.

### 2.3 Materials

*The Existential Concerns Questionnaire* was used to assess existential concerns. This is a 22-item self-report questionnaire, based on the theoretical model of the five factors of existential anxiety: fear of death, meaninglessness, identity, loneliness and guilt (Van Bruggen et al., 2017). Questions are rated as either true or false, with each true and false response scored as one and zero, respectively. The obtainable score ranges from 0 to 22. Higher scores indicate greater existential concerns and lower scores indicate lesser existential concerns. The questionnaire has good internal consistency with a Cronbach's alpha of 0.82 and test-retest reliability of 0.78 (Ain and Gilani, 2021).

*The Community Integration Questionnaire* (Willer et al., 1994) was used to measure community integration. It is a 15-item self-report questionnaire with 3 domains: home integration (H), social integration (S), and integration into productive activities (P). Items 1–12 are scored on a scale of 0 to 2. Items 13, 14 and 15 are scored individually and then transformed into one variable called the jobschool variable which is scored from 0 to 5. The integration into productive activities domain score is obtained by summing the score of the 12th item and the jobschool variable score. The overall score ranges from 0 to 29. The community reintegration level is classified as: low (0–14), moderate (15–20) and high (21–29). The questionnaire has demonstrated good internal consistency with Cronbach's alpha of 0.86–0.90 and test-retest reliability of 0.80–0.90.

*The Depression subscale of the Hospital Anxiety and Depression Scale (HADS-D)* (Zigmond and Snaith, 1983; Geusgens et al., 2024) was used to assess psychological depression. The HADS-D comprises 7 items that are rated on a 4-point Likert scale from 0 to 3. The total score from all items is used to categorize the level or severity of depression as: non-case (0–7), mild (8–10), moderate (11–15) and severe (16–21). The questionnaire has a Cronbach's alpha of 0.80–0.90; and test-retest reliability of 0.80–0.90 (Stern, 2014).

*The Revised Scoring Tool for the Classification of Socio-economic status in Nigeria* (Ibadin and Akpede, 2021) was used to assess the socio-economic status of the participants. The tool has 3 items: level of education, occupation and income. The minimum annual income is set at ₦360,000 (i.e ₦30,000 per month, which was the minimum wage at the time of this study). Each item is graded on a six-point scale from 1 to 6, where 1 represents the highest level and 6 represents the lowest level of each socioeconomic variable. The total score from all items is divided by three (3) and used to classify the participants' socio-economic status into upper (1–2), middle (3–4), or lower (5–6) socio-economic class. A section for obtaining the socio-demographic and clinical information of participants: age, marital status, limb dominance, time since stroke onset, and side of stroke affectation, was added to the socio-economic status form.

### 2.4 Procedure

Ethical approval was obtained from the University of Ibadan/University College Hospital (UI/UCH) Health Research Ethics Committee (UI/UCH/EC/24/0746). Informed consent was obtained from all eligible participants after the purpose and rationale for the study had been fully explained to them.

#### 2.4.1 Quantitative data collection

The existential concerns questionnaire, the depression subscale of hospital anxiety and depression scale, the community integration questionnaire and the revised scoring tool for the classification of socio-economic status in Nigeria were translated through a forward- backward approach. Copies of the questionnaires were hand-distributed and self-administered by participants in their preferred language. Filled-out questionnaires were also collected by hand. Data collection was from October 2024 to February 2025.

#### 2.4.2 Qualitative exploration of existential concerns

The qualitative study was grounded ontologically in relativism (Österman, 2021) and epistemologically in interpretivism, based on the beliefs that there are multiple realities and that context is important in the understanding of participants' existential concerns (Kivunja and Kuyini, 2017). A Focus group discussion (FGD) was employed for the qualitative study. Six purposively sampled female stroke survivors from the survey sample participated in the FGD to explore their individual experiences and existential concerns. The lead author (OAO), who has experience in conducting qualitative research and has published multiple qualitative studies, served as moderator for the FGD, while the co-author was an observer. There was a notetaker who took minutes of the discussions in addition to the audio recordings. The authors were aware of how their positionality and preconceptions could influence the research process and data interpretation. Therefore, they practice reflexivity and approach the FGD with a commitment to minimize biases, thereby enhancing the credibility and trustworthiness of the findings.

A focus guide (Appendix 1), comprising semi-structured open-ended questions formulated from a review of existing literature was used to guide the discussion. Participants were informed of their rights to withdraw from the study at any stage and were assured of anonymity of the data collected. Informed consent for participation and audio recording was obtained from all participants. The discussion was recorded using the audio recorder of a smartphone. Data saturation was considered reached when no substantially new existential concerns emerged, and themes became recurring among participants. The FGD lasted approximately 60 min.

### 2.5 Data analysis

Quantitative data were analyzed using IBM Statistical Package for Social Sciences (SPSS) Version 23. Descriptive statistics were used to summarize the data. Chi-square test was used to investigate the association between community reintegration and each of the following: existential concerns and psychological depression. Associations between these variables and sociodemographic variables (age and marital status), were also tested with Chi-square test. Fisher's exact test was used to examine the associations between the variables and each of time since stroke onset and socio-economic status. The level of significance was set at 0.05.

Qualitative data were analyzed using interpretative phenomenological analysis (Alase, 2017). This approach is well-suited for exploring the lived experiences of research participants, as it allows them to express themselves in their own way without distortion by the researchers, thereby minimizing researcher bias. The authors are female physiotherapists involved in stroke care, who, through cursory clinical observation, have noted that male stroke survivors often received more social support from their wives and children while female survivors tend to receive minimal social support from their husbands. Acknowledging the potential impact of this bias, authors were careful to ensure it did not influence the data analysis and interpretation process. The audio recording of the discussion was promptly and manually transcribed verbatim by one of the authors (ATO) within 48 h of the FGD, to accurately capture participants' perspectives. The transcripts were then verified by the lead author (OAO). Both authors read the transcripts carefully and multiple times, to familiarize themselves with the data and note emerging patterns. Data was then exported into a Microsoft Excel worksheet for analysis. All comments were entered individually into cells in a column. Each quote was reviewed and assigned to subthemes and themes in different columns using an inductive approach. Through a thorough process of review, refinement, grouping and regrouping, five themes were generated and presented sequentially to create a trajectory of the existential concerns of female stroke survivors in Nigeria. Representative quotes were used to illustrate each theme.

## 3 Results

The results of both the quantitative and qualitative studies were merged to provide a detailed exploration and a nuanced understanding of the existential concerns of the participants.

### 3.1 Survey results

Seventy-five out of the 78 copies of the questionnaires administered to participants were properly completed and analyzed, giving a response rate of 96.2%. Participants were aged 64.07 ± 14.03 years, with a mean time since stroke onset of 47.23 ± 63.97months. More than half (58.7%) were in the lower socio-economic stratum. The mean community reintegration, existential concerns and psychological depression scores were 12.24 ± 2.95, 9.77 ± 5.52, and 13.84 ± 4.71, respectively. The socio-demographic and clinical characteristics of the participants are as summarized in Table 1.

**Table 1:** Socio-demographic and clinical characteristics of participants (*N* = 75).

**Variables**	**Frequency (*n*)**	**Percentage (%)**
**Age (years)**		
20–29	2	2.7
30–39	3	4.0
40–49	10	13.3
50–59	16	21.3
60–69	22	29.4
70–79	16	21.3
80–89	6	8.0
Mean ± SD	64.07 ± 14.03	
**Marital status**		
Single	5	6.7
Married	52	69.3
Divorced	2	2.7
Widowed	14	18.6
Separated	2	2.7
**Level of education**		
Postgraduate	1	1.3
First degree/HND	16	21.3
NCE/ND/A'Level GCE	11	14.7
Secondary school certificate/NECO	23	30.7
Primary/modern III/JSS3 certificate	13	17.3
No formal education/non-literate	11	14.7
**Socio-economic status**		
Low	44	58.7
Middle	31	41.3
Upper	0	0.0
**Side of stroke affectation**		
Right	39	52.0
Left	36	48.0
**Time since stroke onset (months)**		
Less than 12	25	33.3
12–24	11	14.7
Greater than 24	39	52.0
Mean ± SD	47.23 ± 63.97	

Community reintegration was generally poor. The majority (81.3%) of the participants had low reintegration scores. Fifty-nine (78.7%) participants seldomly leave their homes, and 72.0% rely on others for shopping (Table 2). Most of the participants (62.7%) reported moderate existential concerns (Table 2). More than two-thirds (69.3%) reported a persistent sense of threat, while 57.3% struggled with loneliness and 74.7% struggled with a loss of meaning (Table 3). More than half of the participants (57.3%) were classified as having no depression (Table 2), though 34 (45.3%) reported being cheerful only sometimes.

**Table 2:** Existential concerns, community integration and psychological depression among participants (*n* = 75).

**Variables**	**Frequency (*n*)**	**Percentage (%)**
**Existential Concerns**		
Low	16	21.3
Moderate	47	62.7
High	12	16.0
**Community Integration**		
Low	61	81.3
Moderate	14	18.7
High	0	0.0
**Psychological Depression**		
Normal	43	57.3
Mild	14	18.7
Moderate	13	17.3
Severe	5	6.7

**Table 3:** Summary distribution of responses to existential concerns (*N* = 75).

**Questions**	**Yes *n* (%)**	**No *n* (%)**
1. The question of whether life has meaning makes me anxious	35 (46.7)	40 (53.3)
2. It frightens me when I realize how many choices life offers	19 (25.3)	56 (74.7)
3. I worry about not being at home in the world, as if I do not belong here	36 (48.0)	39 (52.0)
4. Existence feels threatening to me, as if at any moment something terrible could happen to me	52 (69.3)	23 (30.7)
5. It frightens me that at some point in time I will be dead	33 (44.0)	42 (56.0)
6. I worry about the meaning of life	23 (30.7)	52 (69.3)
7. I try to forget that all my choices have consequences	23 (30.7)	52 (69.3)
8. I get anxious because of losing touch with myself	37 (49.3)	38 (50.7)
9. I struggle with the feeling that in the end I am on my own in life	43 (57.3)	32 (42.7)
10. It makes me anxious that my life is passing by	27 (36.0)	48 (64.0)
11. When the question of whether life has meaning enters my mind, I try to think quickly about something else	46 (61.3)	29 (38.7)
12. I worry about not living the life that I could live	46 (61.3)	29 (38.7)
13. The awareness that other people will never know me at the deepest level frightens me	45 (60.0)	30 (40.0)
14. I worry that, out of the blue, something terrible might happen to me	36 (48.0)	39 (42.0)
15. I try to push away the thought that life will end	24 (32.0)	51 (68.0)
16. It frightens me that things I once considered important seem meaningless when I look back on them	56 (74.7)	19 (25.3)
17. I am afraid that I do not get out of life what is in it	35 (46.7)	40 (53.3)
18. I try to avoid the question of who I really am	23 (30.7)	52 (69.2)
19. I have the anxious feeling that there is a gap between me and other people	50 (66.7)	5 (33.3)
20. I become anxious when I realize how vulnerable my body is to the dangers of life	50 (66.7)	25 (33.3)
21. I worry about having to let go of everything at the moment of my death?	25 (33.3)	50 (66.7)
22. I am afraid that I will never know myself at the deepest level	26 (34.7)	49 (65.3)

There was no significant association between community reintegration and existential concerns (*p* > 0.05) (Table 4). However, a majority of the women with moderate existential concerns reported low community reintegration. There was a significant association between community reintegration and depression (*p* = 0.019), and between existential concerns and psychological depression (*p* < 0.05). There was a significant association between existential concerns and age (p=0.004), whereas there was no significant association between existential concerns and marital status, socio-economic status or time since stroke onset (*p* > 0.05) (Table 5). There was a significant association between psychological depression and marital status (*p* = 0.02).

**Table 4:** Association between existential concerns and each of community integration and psychological depression (*n* = 75).

**Existential concerns**	**Low**	**Moderate**	**High**	**Total**	**χ^2^**	***p*-value**
**Community integration**						
Low	10	40	11	61		
Moderate	6	7	1	14	**5.023**	**0.081**
High	0	0	0	0		
**Psychological Depression**						
Normal	30	13	0	43		
Mild	14	0	0	14	**9.991**	**0.019** ^*^
Moderate	13	0	0	13		
Severe	4	1	0	5		

^*^Significant at *p* < 0.05. The bold values are the chisquare and *p*-values.

**Table 5:** Association between existential concerns, and selected socio-demographic and clinical variables.

**Existential concerns**	**Low**	**Moderate**	**High**	**Total**	**χ^2^**	***p*-value**
**Age**						
20–49	1	7	7	**15**	**15.52**	**0.004** ^*^
60–69	10	23	5	**38**		
70–89	5	17	0	**22**		
Total	**16**	**47**	**12**	**75**		
**Marital status**						
Married	13	33	6	**52**	**2.79**	**0.247**
Unmarried	3	14	6	**23**		
Total	**16**	**47**	**12**	**75**		
					**Fisher**	
**Socio-economic status**						
Low	8	31	5	**44**	**–**	**0.089**
Middle	8	16	7	**31**		
Upper	0	0	0	**0**		
Total	**16**	**47**	**12**	**75**		
**Time since stroke onset**						
< 12 months	5	12	8	**25**		**0.053**
12–24 months	1	8	2	**11**		
>24 months	10	27	2	**39**		
Total	**16**	**47**	**12**	**75**		

^*^Significant at *p* < 0.05. The bold values are the total of participants in each categories.

### 3.2 Exploration of existential concerns among participants

To deepen the understanding of existential concerns among female stroke survivors, a focus group discussion (FGD) was conducted with six participants aged 54.00 ± 7.24 years. To ensure anonymity, participants were identified by numbers (1, 2, 3, 4, 5, and 6) assigned by the researchers. Five emergent themes reflected the existential concerns of female stroke survivors.

#### 3.2.1 Stroke as a devastating condition

The survey findings showed high existential threats among participants. This was echoed in the FGD, where participants described stroke as a devastating and life altering-experience. A participant noted that “*stroke is a terrible experience and a devourer*” (P4, 61years) that drains resources and disrupts life. Others emphasized the suddenness of onset, reinforcing feelings of unpredictability and loss of control:

“*It was very sudden… I just fell down” (P5, 48 years)*

#### 3.2.2 Role disruption and loss of autonomy in the home

Consistent with the survey's findings of low community reintegration, the women lamented their inability to perform house chores or nurture their families. One explained:

“*I have not been able to cook since it happened to me*” (P3, 48years).

Some participants expressed frustrations at being unappreciated despite attempts to contribute:

“*If I cook, my husband won't eat it because I didn't use both hands to make it*. (P4, 61 years)

Another described sneaking to perform house chores “to avoid feeling redundant” (P6, 65 years).

This highlights how stroke undermined the women's valued identities as caregivers, deepening existential distress.

#### 3.2.3 Isolation and stigmatization in society

Survey findings showed that most women seldom left their homes, and the FGD findings provided insights on why. Participants described withdrawing from social events due to perceived stigma and negative attention. One participant stated:

“*I have stopped attending church services…I dislike the way people just stare at me*” (P6, 65 years).

Others felt excluded by neighbors or relegated within community groups. Feelings of being avoided or disregarded led to self-imposed withdrawal, contributing to loneliness and underscoring stigma as a key barrier to reintegration:

“*I go out to parties, but most times, I don't like how people treat me at such events…this discourages me from going out subsequently in order to avoid such experiences*.” (P4, 61 years).

“*Some people don't even associate with me again because of my condition.”* (P1, 52 years).

#### 3.2.4 Inadequate spousal support and changes in sexual intimacy

Emotional struggles experienced by participants were reflected in their narratives of strained spousal support and diminished sexual intimacy. While some couples mutually agreed to suspend sexual intimacy pending recovery, others reported dwindling care and support over time:

“*We both agreed that we won't be sexually intimate until I'm back to good health…”* (P5, 48 years).

“*My husband no longer drives me to the hospital for physiotherapy, this makes me sad. It's my children who help now”* (P1, 52 years).

This erosion of spousal support heightened fears of abandonment and contributed to psychological distress.

“*I told my husband, if he leaves me at this stage…. You know sometimes, he gets angry and I plead with him to stay, he agrees”* (P5, 48 years).

#### 3.2.5 Work-related and financial concerns

Participants described workplace discrimination and financial burdens, aligning with the high prevalence of existential concerns. One woman recounted being pressured at work despite limitations, threatened with redundancy and outright loss of income:

“*My boss would threaten to declare me redundant, which was very painful. I was worried I would be laid off unceremoniously because of my condition”* (P4, 61 years).

The women expressed a desire for financial independence and early return to paid employment:

“*It is my husband that helps me out financially… Is there a way for us to return to our work on time*?” (P5, 48 years).

The participants also decried the lack of insurance coverage for long-term physiotherapy, exacerbating their financial strain.

## 4 Discussion

This mixed-methods study explored existential concerns, depression and community reintegration among female stroke survivors in Ibadan, Nigeria,. By merging quantitative and qualitative findings, we gained a nuanced understanding of how stroke disrupts identity, social roles and psychological wellbeing. There was a notable convergence in our findings.

### 4.1 Existential anxiety and depression

Consistent with previous reports (Oyewole et al., 2016; Badaru et al., 2015), participants described stroke as devastating, unpredictable and life-altering. Quantitative data showed that the majority felt their existence was under constant threat, while qualitative narratives revealed hidden despair and fear of mortality. Many respondents in the quantitative study avoided questions about the meaning of life, possibly as a defense mechanism against deep existential contemplation. It has been suggested that individuals facing major health crises suppress distressing thoughts to maintain emotional stability (Akosile et al., 2011). Despite more than half reporting no depression, the FGD uncovered episodes of crying and hopelessness, suggesting underreporting or social desirability bias (Bakare et al., 2024).

The strong association between existential concerns and depression reflects the interplay among uncertainty, loss of purpose and perceived lack of control (Agbola et al., 2020). Qualitative data highlighted the role of strained intimacy and inadequate spousal support in the prevalence of depression among married women. This reinforces evidence that psychosocial factors are as critical as physical disability in shaping post-stroke depression (Ibeneme et al., 2017).

### 4.2 Social expectations and role reversal

Loss of autonomy in household and caring roles was a central theme. Many women struggled with dependence, secrecy around chores and unappreciated efforts, reflecting how cultural expectations of women as caregivers exacerbate distress when they become care recipients (Vincent-Onabajo et al., 2018; Ogunlana et al., 2023). This has led some women to overextend themselves, resulting in injuries, and increased emotional distress. The perceived lack of appreciation and/or rejection by spouses deepened emotional pain, buttressing findings that unmet social expectations worsen psychological outcomes (Ihegihu et al., 2024). Tailored family education programs are essential to reduce role reversal trauma and validate the contributions of female stroke survivors within households.

### 4.3 Social isolation and stigma

Over half of the survey respondents reported fears about being alone, while FGD highlighted avoidance of public gathering and feeling of exclusion by neighbors and social networks. This aligns with earlier findings that social isolation is a barrier to reintegration (Obembe et al., 2010; Vincent-Onabajo et al., 2015). Stigma, rooted in misconception of stroke as contagious, compounded loneliness and reduced participation (Vincent-Onabajo et al., 2015). Involving survivors in peer-support groups and structured community activities could provide a sense of belonging, reduce stigma and buffer against depressive symptoms.

No significant association existed between existential concerns and community reintegration. This could be due to underreporting of existential distress or the stronger influence of stigma and physical disability on reintegration. However, the significant associations between existential concerns, age and depression highlight how psychological and social contexts shape reintegration trajectories.

### 4.4 Workplace reintegration and financial concerns

Many of the women in our study belonged to the lower socio-economic stratum, and narratives revealed financial strain and workplace discrimination. These findings resonate with reports by Soeker and Olaoye (2017) that stroke survivors in Nigeria struggle to resume work due to physical limitations and workplace discrimination. This underscores the need for policies that support workplace reintegration, which could mitigate barriers and support female stroke survivors' economic independence.

## 5 Study limitations

This study has some inherent limitations. Survey data are based on self-report questionnaires that might have introduced social desirability and recall biases. Also, the FGD sample was small and recruited from a single study site, limiting the generalizability of qualitative findings. Lastly, the cross-sectional design of the quantitative study precludes causal inference. Despite these limitations, the mixed-methods approach adopted for the study provided valuable complimentary perspectives.

## 6 Conclusion

The outcomes of this study highlight the unique existential concerns of female stroke survivors in Nigeria and their links with depression and limited community reintegration. These findings emphasize the need for gender-sensitive, community-based rehabilitation programs that integrate functional recovery with counseling and existential support. Family caregiver education on how to ease role-reversal trauma should be prioritized. Peer-support networks to reduce isolation and stigma should be encouraged for female stroke survivors. Policies to strengthen workplace reintegration, expand health insurance to cover long-term physiotherapy, and embed mental health services within stroke care are urgently needed for this population. Future longitudinal studies to capture changes in existential and psychological outcomes over time are needed. Also, comparative studies to explore how gender differences shape post-stroke adjustment should be conducted to guide the design of equitable and culturally sensitive interventions that could improve the quality of life of female stroke survivors in Nigeria and similar contexts.

## Data Availability

The raw data supporting the conclusions of this article will be made available by the authors, without undue reservation.

## Appendix—Focus Guide

1. Can you kindly share with us what stroke meant to you based on your personal experience of stroke?
2. How has having a stroke affected your roles at homes, at work and your immediate community?
3. In what ways do you feel stroke has changed your identity or self-worth as women?
4. How do you think having a stroke has changed how others see you or what they expect of you? Probe: How do you respond to these changes?
5. What has been your experience in terms of support from your husband, children and other relatives since your stroke? Probe: How has your husband and family encouraged you to return to your previous activities?
6. How has stroke affected your sexual life or intimacy with your husband?
7. Is there anything you will like to tell or ask us that we have not talked about on how stroke has affected you as women?
